# Acute spasticity in malignant MCA stroke: a case report and review of literature

**DOI:** 10.1093/omcr/omaf114

**Published:** 2025-07-27

**Authors:** Muhammad Faizan, Azza Halfawi, Alreem Alkuwari, Mohamad Ali, Mohamed Elgassim, Shahzad Anjum

**Affiliations:** Emergency Medicine Department, Hamad General Hospital, Trauma and Emergency Center, Al Rayyan Road, Doha, Doha Municipality, 3050, Qatar; Emergency Medicine Department, Hamad General Hospital, Trauma and Emergency Center, Al Rayyan Road, Doha, Doha Municipality, 3050, Qatar; College of Medicine, Qatar University, Al Tarfa, Doha, Doha Municipality, 2713, Qatar; Emergency Medicine Department, Hamad General Hospital, Trauma and Emergency Center, Al Rayyan Road, Doha, Doha Municipality, 3050, Qatar; Emergency Medicine Department, Hamad General Hospital, Trauma and Emergency Center, Al Rayyan Road, Doha, Doha Municipality, 3050, Qatar; Emergency Medicine Department, Hamad General Hospital, Trauma and Emergency Center, Al Rayyan Road, Doha, Doha Municipality, 3050, Qatar

**Keywords:** malignant MCA infarction, acute spasticity, ischemic stroke, cerebral edema, anabolic steroids, decompressive craniectomy, stroke risk factors, case report

## Abstract

Malignant Middle cerebral artery (MCA) infarction is characterized by rapid neurological deterioration due to cerebral edema. Spasticity, a common sequela of stroke, typically occurs days to weeks after the initial event. However, an acute onset of spasticity at presentation is extremely rare. We report a case of a 40-year-old male patient who presented with spasticity accompanied by aphasia and decreased consciousness. Left MCA occlusion was demonstrated on Computed tomography (CT) angiography. Cerebral edema developed rapidly, requiring decompressive hemicraniectomy (DH). The patient was a known smoker with androgenic anabolic steroid use. This case represents a rare presentation of malignant MCA infarction with acute spasticity and adds a unique aspect to the clinical spectrum of stroke presentation. This case emphasizes the need for awareness of atypical stroke presentation and the potential role of anabolic steroids in stroke risk.

## Introduction

Malignant middle cerebral artery (MCA) infarction is a devastating form of stroke, with an incidence of 10–20 per 100 000 people per year [1]. Cerebral edema typically develops for 2 to 4 days following an ischemic stroke, but when it involves the entire MCA territory, neurological decline occurs rapidly within 24 h of symptom onset; hence, it is termed ‘malignant MCA infarction’. 25% of patients with stroke develop spasticity during the first six weeks after stroke onset [2]. However, spasticity presenting acutely in the setting of acute thrombotic stroke is unusual, with very few cases reported in the literature.

In this case report, we present a rare case of malignant MCA infarction that was presented with spasticity as its initial symptom. To our knowledge, this is one of the few cases reported in literature.

## Case presentation

**Figure 1:**
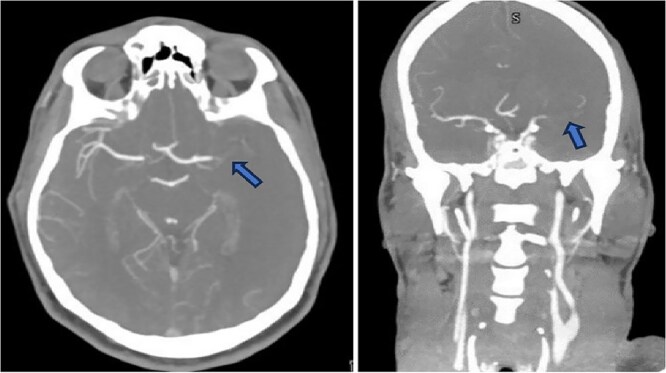
CT angiogram intracranial shows left MCA occlusion at the level of M1 segment. Rest of the anterior and posterior cerebral circulation appear opacified by contrast and is normal in caliber.

**Figure 2:**
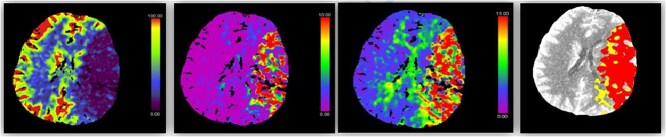
CT brain perfusion study shows a large area of matched perfusion defect in left frontotemporal parietal lobes representing infarct core with surrounding penumbra is noted.

**Figure 3:**
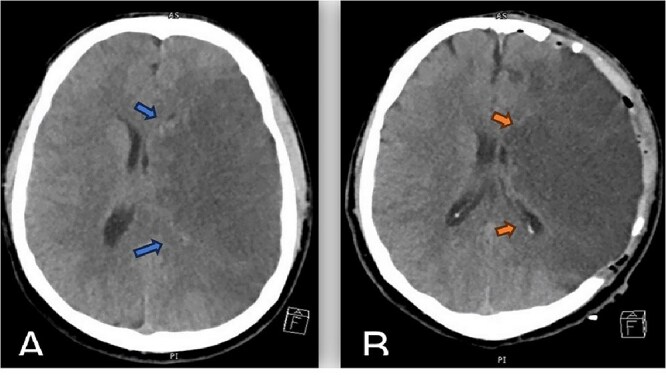
(A) CT head shows interval development of a right sided midline shift of 6.8 mm causing effacement of the left lateral ventricle. (B) Post decompressive craniectomy CT head shows interval decrease of a right sided midline shift, measuring 1.5 mm (previously 6.8 mm), with mild re-expansion of the left lateral and third ventricles.

**Figure 4:**
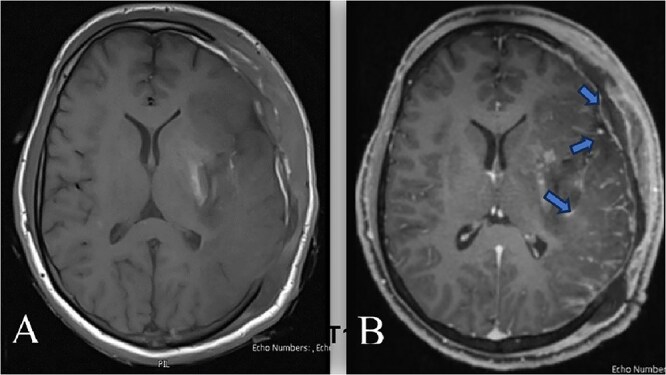
Post- craniectomy T1 weighted MRI head axial view. (A) Pre- gadolinium contrast (B) post- gadolinium contrast: Postcontrast left hemispheric prominent superficial vascular and leptomeningeal enhancement.

A 40-year-old previously healthy male patient, known smoker (20 cigarettes per day) with a history of testosterone injection cycles (unsupervised, dosage unknown), brought by emergency medical staff due to a decreased level of consciousness. He was last seen active 5 h prior. Neurological examination revealed a global Glasgow Coma Scale (GCS) score of 9 (E3 M6 V1), with a National Institutes of Health Stroke Scale (NIHSS) score of 20. There was right-sided central facial palsy and hemiplegia (power 3/5 in the right upper and lower limbs). Babinski sign was observed unilaterally on the left side. Computed tomography (CT) angiography of the head revealed left MCA occlusion at the level of the M1 segment without a midline shift (Fig. 1). CT perfusion showed a large area of matched perfusion defects in the left frontotemporal parietal lobes, representing large infarct core with minimal surrounding penumbra (Fig. 2). Therefore, thrombolysis and thrombectomy were unobtainable. The initial blood work up and ECG findings were unremarkable. The patient was subsequently admitted to the ICU for further care. 48 h later, the patient was noted to be drowsier, GCS score dropped from 9 to 8 [E3 M4 V1]. Repeated neuroimaging showed a midline shift of 6.8 mm causing effacement of the left lateral ventricle (Fig. 3A) suggesting cerebral edema. A lifesaving decompressive craniectomy was performed within 6 h of the GCS drop. Post-decompressive craniotomy imaging showed an interval decrease of a right-sided midline shift, measuring 1.5 mm (previously 6.8 mm), with mild re-expansion of the left lateral and third ventricles (Fig. 3B). The post-operative course was complicated by increased levels of inflammatory marker CRP: 286.3 mg/l (normal: < 5.0 mg/l) and WBC: 15.9 × 10^3^/μl (normal: 4.0–10.0 × 10^3^/μl), raising the probability of meningitis. Lumbar puncture (LP) was not performed due to the patient being on aspirin and heparin, which increased the risk of bleeding complications. T1 weighted contrast Magnetic resonance imaging (MRI) showed prominent left hemispheric superficial vascular and leptomeningeal enhancement, which can also be attributed to the early subacute phase of the infarction; however, underlying meningitis could not be completely excluded (Fig. 4A and B). He was administered 4.5 g of piperacillin/tazobactam every 8 h for 3 days, followed by 1.25 g of vancomycin every 12 h for the next 2 days, and then 2 g of meropenem every 8 h for 14 days. Repeated labs 3 days after initiating antibiotic therapy showed improvement of inflammatory markers CRP: 169.9 mg/l, WBC: 9.8 × 10^3^/μl. The cranioplasty was done 3 months after the first presentation. Currently, the patient is conscious, oriented and on regular physiotherapy for his remaining hemiplegia and aphasia.

## Discussion

Cerebrovascular disease is a global cause of death and functional mortality. Ischemic injuries vary widely ranging from clinically silent lesions to fatal infarctions. MCA infarctions have an approximate in-hospital mortality rate of 17% [3]; however, mortality rises to nearly 80% in individuals with malignant MCA infarction [4].

Malignant MCA infarction is a subtype of ischemic stroke that affects over 50% of the MCA territory and can extend into other vascular regions. The cerebral edema’s space occupying effects lead to progressive neurological decline and decreased consciousness in such patients. The term was coined by Hacke et al. in 1996 [5]. Embolic or thrombotic occlusion of the distal internal carotid artery or main MCA branch (M1 segment) is the primary cause and this type of infarction accounts for roughly 10% of all supra-tentorial strokes [4].

Pathophysiology involves compromise of cerebral blood flow which causes the dysfunction of Na + -K+ ATPase pump, hence leading to cytotoxic edema. Ischemia also leads to increased permeability of blood–brain barrier, causing fluid to shift to extracellular compartment and resulting in vasogenic edema typically within three days of symptom onset [6].

In our case, the patient’s GCS dropped 48 h after admission due to the development of significant cerebral edema, causing mass effect and right ventricular effacement as confirmed by repeat imaging. Cerebral edema, apart from being the primary cause of neurological decline in these patients, also increases the risk of brain herniation.

Decompressive hemicraniectomy (DH) is the treatment of choice in such patients, as it allows the brain to expand by increasing cranial cavity volume. This procedure prevents rise in intracranial pressure and herniation, improves cerebral blood flow, and enhances tissue oxygenation. Studies have shown an 80% improvement in mortality with DH, which our patient also underwent to relieve cerebral edema’s mass effect [7, 8].

The uniqueness of our case lies in the acute presentation of spasticity which is considered as an atypical symptom in acute lesions, where flaccidity is usually observed initially. Spasticity following stroke typically develops gradually, between 1–6 weeks post-stroke [2]. A systematic review showed that only 4%–27% of patients develop spasticity within the first week after stroke [9]. A malignant MCA stroke causes extensive cortical and subcortical damage thus resulting in the disruption of inhibitory pathways (e.g. reticulospinal tract), this causes loss of inhibitory control over spinal motor neurons resulting in spasticity [10].

The literature review (Table 1) presents similar reported cases in the literature with comparison between key similarities and differences to this case. While none of the MCA occlusion cases reported spasticity, 2 cases of anterior cerebral artery occlusion reported spasticity as an initial presentation, supposedly related to a pathology affecting the Cingular gyrus. However, this case presents a more distributed insult within the frontotemporal parietal lobe.

**Table 1:** Comparision between similar reported cases in the literature with highlight of key similarities and differences to this case

Author (Year)	Title	Key Similarities	Key Differences
Alzahrani WM et al., (2023) [13]	Acute spasticity secondary to ischemic stroke involving superior frontal gyrus and anterior cingulate gyrus	Spasticity as an initial presentation Previously healthy patient	Saccular aneurysm originating from the anterior communicating artery Involvement of the superior frontal gyrus and cingulate gyrus
Alves I et al.(2013) [14]	Spasticity as the First Manifestation of Ischemic Lesions Involving the Cingulum	Patient 1: Spasticity as an initial presentation Previously healthy patient (non-smoker) Patient 2: Spasticity as an initial presentation Previously healthy (smoker)	Ischemic defect of pericallosal artery a terminal branch of ACA* Involvement of the superior frontal and cingulate gyri Ischemic defect of pericallosal artery a terminal branch of ACA Involvement of the right frontal lobe, extending to the right cingulate gyrus
Habes et al. (2024) [15]	Stroke as an unusual initial presentation of ‘malignant’ middle cerebral artery infarction involvement in systemic lupus erythematosus	The patient developed massive infarction in the MCA^a^ region complicated to malignant MCA syndrome Previously healthy young patient	Spasticity was not reported The patient was found to have SLE^b^
Jacob et al. (2022) [16]	Malignant middle cerebral artery syndrome with thrombotic thrombocytopenia following vaccination against SARS-CoV-2	The patient developed massive infarction of the M1 segment of MCA complicating into malignant MCA syndrome	Spasticity was not reported The patient was diagnosed with vaccine-induced thrombotic thrombocytopenia leading to arterial thrombosis
Moughal et al. (2021) [17]	Malignant middle cerebral artery infarction following subacute subdural hematoma: A case report and literature review	The patient developed massive infarction in the MCA region complicated to malignant MCA syndrome Previously healthy patient	Spasticity was not reported The patient developed right hemispheric acute subdural hematoma

^a^Middle cerebral artery

^b^Systemic lupus erythematosus

^c^Anterior cerebral artery

In our case the patient was a previously healthy young adult with a history of smoking and anabolic steroid use. Anabolic steroids significantly increase the risk of cardiovascular and cerebrovascular events through several mechanisms. They induce hypercoagulability by elevating clotting factors, increasing serum homocysteine, and can lead to vascular vasospasm by inhibiting the smooth muscle relaxing effect of nitric oxide [11, 12]. Considering these effects, this patient’s history of steroid use may have compounded risk factors, potentially precipitating his malignant stroke.

## Conclusion

This case highlights the importance of vigilant clinical observation and the ability to interpret evolving neurological signs in the management of acute stroke. The early onset of spasticity coupled with neurological deterioration served as critical indicators of impending malignant edema. Recognizing these atypical yet significant signs allowed for prompt neuroimaging and timely surgical intervention. The case exemplifies how attentive clinical evaluation when integrated with instrumental diagnostics helps in early detection of life-threatening complications.

## Research registration

Not done (no new equipment/technology used).
